# Understanding the Wnt Signaling Pathway in Acute Myeloid Leukemia Stem Cells: A Feasible Key against Relapses

**DOI:** 10.3390/biology12050683

**Published:** 2023-05-05

**Authors:** Daniel Láinez-González, Ana Belén Alonso-Aguado, Juan Manuel Alonso-Dominguez

**Affiliations:** 1 Experimental Hematology, Instituto de Investigación Sanitaria Fundación Jiménez Díaz, Universidad Autónoma de Madrid, Avenida Reyes Católicos 2, 28040 Madrid, Spain; daniel.lainez@fjd.es (D.L.-G.);; 2 Hematology Department, Hospital Universitario Fundación Jiménez Díaz, Avenida Reyes Católicos 2, 28040 Madrid, Spain

**Keywords:** HSCs, LSCs, AML, quiescence, hematopoiesis, β-catenin, Hedgehog, Notch

## Abstract

**Simple Summary:**

In hematological malignancies, specifically in acute myeloid leukemia, aberrant stem cells, also known as leukemic stem cells, may be responsible for the relapse of the disease. Since several authors have related the quiescence and chemoresistance of leukemic stem cells with the Wnt/β-catenin signaling pathway, new approaches to chemosensitize this population should be studied. Therefore, in this review, we summarize the current information about the Wnt/β-catenin signaling pathway in hematology.

**Abstract:**

Wnt signaling is a highly conserved pathway in evolution which controls important processes such as cell proliferation, differentiation and migration, both in the embryo and in the adult. Dysregulation of this pathway can favor the development of different types of cancer, such as acute myeloid leukemia and other hematological malignancies. Overactivation of this pathway may promote the transformation of pre-leukemic stem cells into acute myeloid leukemia stem cells, as well as the maintenance of their quiescent state, which confers them with self-renewal and chemoresistance capacity, favoring relapse of the disease. Although this pathway participates in the regulation of normal hematopoiesis, its requirements seem to be greater in the leukemic stem cell population. In this review, we explore the possible therapeutic targeting of Wnt to eradicate the LSCs of AML.

Acute myeloid leukemia (AML) is a group of hematopoietic neoplasms characterized by an increase in myeloid blasts. However, AML exhibits a high degree of molecular heterogeneity, as recently recognized in the new diagnostic classifications [1,2]. AML shows a hierarchical organization with leukemic stem cells (LSCs) at its apex. LSCs are thought to be resistant to conventional chemotherapy and be the origin of the high rate of relapses observed in AML. In this review, we will explore the role of Wnt signaling on hematopoietic neoplasms with special emphasis on AML and LSC.

## 1. Wnt Signaling Pathway

In 1980, mutations in the *wingless* (*wg*) gene of *Drosophila melanogaster* were found to produce aberrant embryonic development [3]. Later, in 1982, the proto-oncogene *Int-1* (*Wnt1*) was identified in mice as an homologue of *wg* [4]. This was the beginning of multiple studies that revealed the existence of an evolutionarily conserved signaling pathway involved in development, organogenesis and oncogenesis [5,6,7,8,9].

Wnt proteins act as morphogens, inducing cell differentiation processes during embryonic development [10]. In humans, there are 19 Wnt-related genes, as far as is known [11]. Wnt ligands can activate three signaling cascades: one canonical (Wnt/β-catenin) and the other two non-canonical (Planar Cell Polarity [PCP] and Wnt/Ca^2+^). The process of Wnt maturation and secretion begins in the endoplasmic reticulum (ER) [12,13]. Modified Wnt ligands interact with the transmembrane protein WLS/EVI in the Golgi apparatus to be translocated to the plasma membrane [14,15]. Wnt proteins can then leave the cell directly and solubilize in the extracellular medium [16], in exosomes [10] or lipoproteins [17].

**Figure 1:**
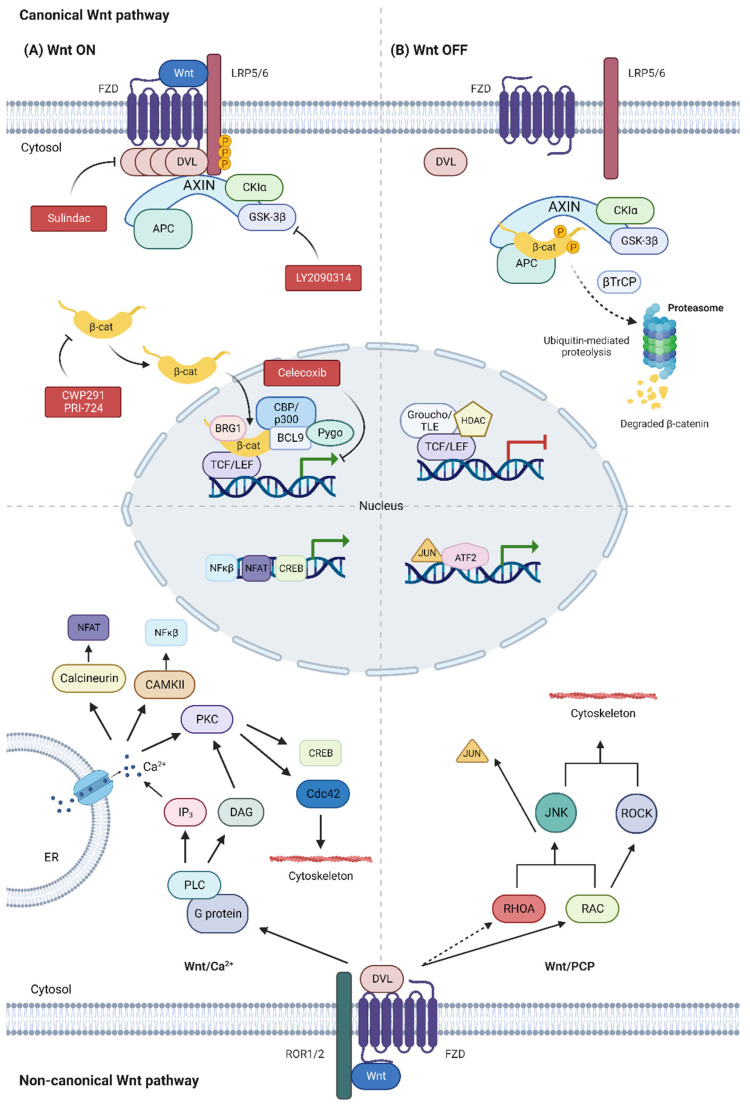
**Canonical Wnt pathway**. (**A**) In the canonical Wnt/β-catenin pathway, binding of the Wnt ligand to the FZD receptor and co-receptor LRP5/6 promotes phosphorylation of LRP5/6 by CKIα and GSK3β kinases and polymerization of the DVL protein. Therefore, the destroyer complex is inactivated (Axin, APC, CKIα and GSK3β) and the β-catenin protein is accumulated in the cytosol. The β-catenin translocates to the nucleus and interacts with the TCF/LEF complex, promoting the recruitment of transcriptional co-activators (CBP/p300, BRG1, BCL9 and Pygo), inducing the expression of target genes. (**B**) In the absence of the Wnt ligand, the destructor complex phosphorylates and induces ubiquitination of β-catenin by βTrCP for its degradation in the proteosome. The absence of nuclear β-catenin favors the recruitment of HDACs by the TCF/LEF and Groucho/TLE repressor complexes, thus inhibiting the expression of target genes. **Non-canonical Wnt pathway**. Non-canonical Wnt ligands can activate other non-canonical β-catenin-independent signaling pathways, such as the Wnt/PCP and Wnt/Ca^2+^. In the Wnt/Ca^2+^ pathway, the Wnt ligand binds to FZD and ROR1/2 and promotes PLC activation via G proteins in a DVL-dependent way. PLC catalyzes the formation of IP3 and DAG, which results in an increase of Ca^2+^ in the cytosol and activation of kinases such as PKC, Calcineurin and CAMKII. These kinases can modify the cytoskeleton through the small GTPase CDC42 and/or regulate transcription of target genes through NFAT, NFκβ and CREB, among others. In the Wnt/PCP pathway, binding of the Wnt ligand to the FZD receptor and co-receptors, such as ROR1/2, favors the activation of small GTPases of the Rho family (RHOA, RAC) by DVL. RHOA and RAC activate JNK kinases and ROCK kinases, which are involved in the reorganization of the cytoskeleton and/or activation of gene expression via Jun/ATF2, among others. Dashed arrows indicate an indirect activation.

In the canonical Wnt/β-catenin pathway, the absence of Wnt ligands subserves phosphorylation of the effector protein β-catenin by the destruction complex. This complex is formed by APC, Axin and two Ser/Thr kinases (CK1α and GSK3β). Phosphorylated β-catenin will be then ubiquitinated by β-TrCP and degraded in the proteosome. In the absence of nuclear β-catenin, TCF/LEF and TLE/Groucho complexes recruit histone deacetylases (HDACs) that repress expression of target genes. Binding of secreted Wnt proteins (e.g., WNT1, WNT3A) to the Frizzled receptor (FZD) and receptor-related protein 5/6 (LRP5/6), which act as coreceptors, promotes phosphorylation of LRP5/6 by CK1α and GSK3β kinases. This phenomenon induces the recruitment and activation of Dishevelled protein (DVL), which binds to FZD and polymerizes. DVL polymers inactivate the destruction complex, resulting in the stabilization and accumulation of β-catenin in the cytosol. Finally, β-catenin translocates to the nucleus, where it will form a complex with TCF/LEF proteins to recruit transcriptional co-activators (e.g., CBP/p300, BRG1, BCL9 and Pygo) and activate the expression of target genes [18,19] (Figure 1).

Most of the target genes of the canonical Wnt pathway are cell-type specific and can promote cell proliferation, but also control postmitotic cell fate and differentiation [20]. Some of them are involved in the maintenance of cancer stem cells (CSCs), such as *MYC* (Myc proto-oncogene protein), *CCND1* (CyclinD1) and *ABCB1* (ABC multidrug transporter) [21].

As mentioned, some non-canonical Wnt ligands (e.g., WNT5A, WNT11) can activate non-canonical β-catenin-independent pathways, such as Wnt/PCP and Wnt/Ca^2+^ (Figure 1). These non-canonical pathways mainly regulate cell migration and adhesion processes; their overactivation can promote epithelial–mesenchymal transition and survival of CSCs [22,23].

The Wnt/PCP pathway is usually initiated when non-canonical Wnt ligands bind to FZD and associated co-receptors, such as receptor tyrosine kinase-like ROR1/2. This binding recruits cytosolic adaptor proteins, such as DVL, which activates small GTPases of the Rho family (e.g., RHOA and RAC). These GTPases activate ROCK and JNK kinases, resulting in cytoskeleton modifications and/or transcriptional responses through c-Jun protein (JUN) activation by JNK and ATF2 recruitment, among others [24] (Figure 1).

The Wnt/Ca^2+^ pathway is activated by the binding of non-canonical Wnt ligands to FZD receptors and co-receptors such as ROR1/2, which induces the recruitment of DVL and the activation of phospholipase C (PLC) by G proteins. PLC transforms phosphatidylinositol 4,5-bisphosphate into diacylglycerol (DAG) and inositol triphosphate (IP3). IP3 promotes the outflow of Ca^2+^ from the ER to the cytosol, whose increase favors the activation of protein kinase C (PKC), calcium/calmodulin-dependent protein kinase type II (CAMKII) and calcineurin phosphatase. The activation of these proteins derives in Ca^2+^-dependent cytoskeleton responses through the small GTPase Cdc42 and/or changes in gene transcription through NFAT, NFκβ and CREB, among others [18,19,25] (Figure 1).

## 2. Wnt Signaling in Normal Hematopoiesis

Hematopoietic stem cells (HSCs) are multipotent cells with self-renewal capacity, precursors of blood cells of the myeloid and lymphoid lines. HSCs reside in the adult bone marrow, where they are usually in a quiescent state in a specialized stroma that regulates their migration and differentiation [26]. However, in the fetus, the liver is the main hematopoietic organ, where HSCs have a high proliferation capacity [27].

Initially, Wnt ligands were described as growth factors (mainly WNT5A) that induced HSC proliferation in in vitro assays [28,29]. Later, other studies suggested the importance of the canonical Wnt pathway in the maintenance of HSCs, since its inhibition with *Dkk1* disrupted the quiescence state of HSCs or the deficiency of Wnt3a disrupted the self-renewal capacity of the same population [30,31]. The same results were obtained when Wnt signaling was inhibited by overexpressing Wnt-inhibitory factor 1 (*Wif1*) in osteoblasts; a study in which it was also observed that the deregulation of two other pathways, such as Hedgehog and Notch, was involved in the maintenance of HSCs [32]. This fact highlights the crosstalk established between these three pathways. Another study showed that Wnt activation by overexpression of WNT3A in stromal cells induced B cell dedifferentiation, whereas the non-canonical WNT5A ligand produced the opposite effect [33], suggesting the possible reversibility of early differentiation stages in lymphopoiesis and the involvement of the Wnt pathway in this process.

However, several Wnt gain-of-function studies showed contradictory results. Some of them proposed that activation of the canonical Wnt pathway with WNT3A or by retroviral expression of a constitutively active form of β-catenin favored self-renewal of HSCs and their repopulation ability in vivo from irradiated mouse tissues [34,35]. On the other hand, other studies revealed that constitutional activation of the canonical Wnt pathway with a nondegradable form of β-catenin reduced the self-renewal capacity of HSCs and led to failures in hematopoiesis [36,37].

The diversity of the exposed results suggested a possible dose-dependent regulation, which was supported by a study performed with different strains of *Apc* mutant mice. The results revealed that elevated levels of Wnt reduced the repopulating capacity of HSCs, whereas a mild increase in the activity of this pathway favored the maintenance of their stem cell functions [38]. Later, the same group associated the depletion of HSCs at high levels of Wnt with increased differentiation and reduced proliferation of these cells [39].

Although all these studies imply the importance of the Wnt pathway in the regulation of normal hematopoiesis, the requirements appear to be higher in fetal HSCs than in normal adult bone marrow HSCs [40]. This could indicate the potential of the Wnt pathway as a therapeutic target for the treatment of AML and other types of leukemia in which it is deregulated.

## 3. The Role of Wnt/β-Catenin Signaling in AML and Other Hematologic Neoplasia

The Wnt signaling pathway regulates the processes of cell proliferation, differentiation and migration. Therefore, any mutation in the elements of this pathway can favor the development of different types of cancer, such as AML and other hematological malignancies [41,42]. Furthermore, in the previous section, we have reviewed the importance of Wnt on HSCs; here, we will also address their malignant counterpart, leukemic stem cells. LSCs have the same features as a stem cell: self-renewal and differentiation. LSCs seem to be responsible for the relapse of the disease; therefore, it is important to understand the role of β-catenin in this population.

It has been shown that in B-cells of Chronic Lymphocytic Leukemia (CLL), mRNA related to Wnt elements is overexpressed, such as *WNT3*, *WNT5B*, *WNT6*, *WNT10A*, *WNT14*, *WNT16* or *FZD3* [43]. Furthermore, 14% of CLL patients have mutations in the Wnt pathway according to Wang et al. Indeed, this study with human samples also showed that the survival of B-cells of CLL with mutations in elements of the Wnt pathway depended on the activation of this pathway [44]. If we study the B-cells of Acute Lymphoblastic Leukemias (ALL), the accumulation of β-catenin leads to downregulation of C-MYC and upregulation of C-JUN, leading to apoptotic induction and cell cycle arrest [45].

The case in T-cell lymphoblastic leukemias is similar. Two studies carried out in mouse models suggested that activation of β-catenin, together with inactivation of *Pten* and, in this case, overexpression of *C-Myc*, favored expansion of LSCs in NOTCH1-independent T-ALL [46,47]. Meanwhile, several authors highlight the interaction among Wnt signaling and other signaling pathways, such as FOXO3 [48].

In myeloid leukemias, the Wnt signaling pathway also plays an important role. For example, it has been described that the deletion of β-catenin delays the recurrence of chronic myeloid leukemia (CML) after imatinib discontinuation [49]. Nevertheless, β-catenin is not the only important protein of Wnt signaling; targeting DVL protein increases the susceptibility of CML cell lines to imatinib. Nonetheless, the effects of silencing *DVL* are complex, since while reducing the signaling of Wnt/β-catenin and Wnt/PCP signaling, it increases the signaling of Wnt/Ca^2+^ [50]. Finally, it has been proven that β-catenin can be stimulated by BCR-ABL1 during the blast phase of CML. This result shows that the inhibition of β-catenin in combination with inhibitors of the tyrosine kinase may delay the blastic transformation of CML [51].

**Figure 2:**
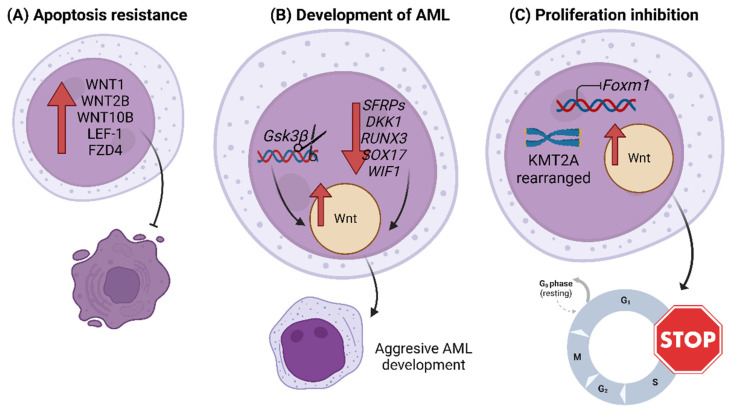
**Studies related to the role of the Wnt/β-catenin signaling pathway in Acute Myeloid Leukemia.** (**A**) In AML, overexpression of ligands, receptors or transcription factors of the Wnt pathway, such as WNT1, WNT2B, WNT10B, FZD4 and LEF-1, entails an antiapoptotic effect. (**B**) Silencing by promoter hypermethylation of Wnt regulatory genes, such as *SFRPs*, *DKK1*, *RUNX3*, *SOX17* or *WIF1*, can activate the pathway. Deletion of *Gsk3β* also activates the Wnt pathway. These actions favor aggressive development of AML. (**C**) The inhibition of *Foxm1* in *KMT2A*-rearranged AML LSCs favors the quiescent state due to the activation of the Wnt signaling pathway.

On the other hand, several studies reported the upregulation of Wnt elements in primary samples of AML patients; some examples are the gene overexpression of ligands (such as WNT1, WNT2B and WNT10B) and transcription factors (such as LEF-1), or the protein overexpression of receptors (such as FZD4) [52,53,54,55]. These overexpressions were associated with increased resistance to apoptosis [52,53] (Figure 2A). The results of one of these studies showed that the expression of a long isoform of LEF-1 predominated in AML cells, whereas normal HSCs expressed a short variant of LEF-1 that lacked a β-catenin binding site [55]. *Gsk3β* regulates Wnt; its deletion increases Wnt signaling in HSCs and favors the development of aggressive AML in mice; therefore, it appears to be involved in the development of hematological malignancies [56] (Figure 2B). The transformation of pre-LSCs into AML LSCs has also been related to overactivation of the Wnt pathway [57,58].

A recent study revealed higher expression of β-catenin protein levels in AML-relapsed samples than in newly diagnosed patients. Furthermore, β-catenin levels were higher in bone marrow samples than in peripheral blood samples, suggesting that the bone marrow microenvironment might induce β-catenin expression in AML cells. They also determined that inhibition of the Wnt/β-catenin pathway with PRI-724 in vitro induced apoptosis in AML cells and suppressed their growth by retaining them in the G1 phase of the cell cycle [59]. Another study showed that *Foxm*1 inhibited the proliferation of mouse and human *KMT2A*-rearranged AML LSCs, favoring their quiescent state and self-renewal through activation of the Wnt/β-catenin pathway, among other molecular pathways. *Foxm1* loss favored the exit of LSCs from the G0 phase and entry into the S phase of the cell cycle, induction of apoptosis and sensitivity to chemotherapy, thus delaying relapse in murine models [60] (Figure 2C).

Finally, several studies have identified Wnt pathway regulatory genes whose silencing by promoter hypermethylation leads to Wnt activation and may contribute to AML development; among them are the antagonists of this pathway, such as *SFRPs*, *DKK1*, *RUNX3*, *SOX17* and *WIF1* [61,62,63]. Treatment with demethylating agents favors the expression of these genes and, therefore, the inhibition of Wnt signaling [62] (Figure 2B). Other epigenetic mechanisms involved in tumor suppression are non-coding RNAs; the expression of MiR-212-5p reduces cell viability and inhibits proliferation in Kasumi-1 cells targeting *FZD5* [64]; while MiR-150, which is related to *FZD4*, is downregulated in AML, ALL and CML, and its expression levels are normalized after complete remission [65].

The value of β-catenin in the LSCs have been studied by several authors in different hematologic neoplasia. Harrison et al. performed experiments on AML to show that the transformation of a progenitor cell to a malignant clone requires the reactivation of β-catenin. Certainly, the study demonstrates that the inhibition of β-catenin results in a reduction of the proliferation and self-renewal of AML cells. In this work, the authors employed NUC-7738, which may reduce this signaling pathway by regulating GSK3β [57]. Nevertheless, Zhao et al. warn that targeting β-catenin could have unexpected effects beyond the purpose of eradicating AML LSCs, since genetic deletion of β-catenin did not affect the ability of LSCs to propagate AML in xenotransplants [66].

Taken together, these studies suggest that overactivation of the Wnt/β-catenin pathway may favor the development and relapses of different hematological malignancies through the maintenance of LSCs. However, further studies are needed to gain a more precise understanding of the molecular mechanisms involved in these processes and to discover new therapeutic targets.

## 4. Wnt/β-Catenin Signaling Pathway as a Therapeutic Target in AML

Several studies have revealed the deregulation of the Wnt pathway in different neoplasms. This is why numerous drugs have been developed to target the elements of this cascade: inhibitors of LRP5/6, DVL and GSK3, antagonists and monoclonal antibodies against FZD receptors or antagonists of the β-catenin/transcription factors interaction (e.g., CBP, TCF, LEF1 and CREB), among others [67,68]. The evaluation of most of these drugs is in the preclinical phase. Some clinical trials have also been initiated, mainly in solid tumors, reaching phases I or II. Nevertheless, these trials lack available results or have reported important adverse effects [68].

Regarding clinical trials in patients with AML (Table 1), most of them employed inhibitors (CWP291, PRI-724 and Celecoxib) or antagonists (Sulindac) of the Wnt/β-catenin pathway to delay tumor progression and prevent relapses, as overactivation of this pathway has been linked to AML progression and chemoresistance of LSCs [57,58,60]. However, the only clinical trial that has reported results (NCT01214603) in refractory or untreated AML patients employed a GSK-3 inhibitor (LY2090314), which produces an increase in β-catenin levels; this agent was well tolerated by patients, but neither complete nor partial tumor remissions were observed [69].

Although all these drugs have a similar goal, they have different targets. CWP291 induces endoplasmic reticulum stress, which activates caspases. These proteins have been revealed to target β-catenin for degradation through a directed cleavage [70]. PRI-724 is another drug related to the inhibition of β-catenin. In this case, PRI-724 disrupts the interaction between β-catenin and CBP [71]. It seems that this drug has low off-targets proteins and it is well tolerated in the clinic [72]; therefore, it could be a good approach to inhibit the Wnt/β-catenin pathway. Finally, Ai et al. studied different compounds, some of them with the possibility not only to inhibit Wnt/β-catenin but providing an anti-leukemia effect at the same time in the CML K562 cell line [73].

Celecoxib, a COX-2 inhibitor, is a non-steroidal anti-inflammatory drug; it has been shown that these kinds of treatments decrease the transcriptional activity of β-catenin [74]. Indeed, COX-2 and its enzymatic product PGE2 have been reported to induce β-catenin expression and an increase in AML cells chemoresistance [75]. Nowadays, one of the recently initiated clinical trials (NCT03878524), which includes Celecoxib, is an umbrella trial that will evaluate the response of patients with various hematological malignancies to different drugs. Another treatment of the family of non-steroidal anti-inflammatory drugs is Sulindac. This approach seems to decrease the non-phosphorylated β-catenin, which is responsible for translocation to the nucleus and starting the transcription of the target genes [76,77].

In this review, it has been explained that Wnt is dispensable for adult hematopoiesis [78]. Henceforth, all these drugs have the potential to achieve different methods of inhibiting the Wnt/β-catenin pathway.

## 5. Discussion

Since 1980, the study of the elements involved in Wnt signaling has revealed the fundamental role of this pathway in cell proliferation, differentiation and migration processes during embryogenesis, as well as tissue renewal in adults [20]. Alterations in some of these highly conserved molecules have been linked to the development and progression of various types of cancer, including hematological malignancies [18,67].

Although the canonical Wnt pathway is involved in the maintenance of normal HSCs, its function appears to be more relevant to and dependent on bone marrow niche signals in LSCs, suggesting the possibility of targeting Wnt without disrupting normal hematopoiesis in patients [40,79]. Several studies have been carried out to evaluate the involvement of this pathway in hematopoiesis. Loss-of-function studies revealed that inhibition of the canonical Wnt pathway disrupts the quiescent state of HSCs, reducing their capacity for self-renewal and tissue regeneration [30,31,32]. However, gain-of-function studies showed non-concordant results [34,35,36,37], suggesting that the effects of the Wnt pathway in hematopoiesis are tightly regulated in a dose-dependent manner [38]. Even so, more studies are needed to precisely understand the mechanisms by which the canonical Wnt pathway regulates these processes.

Overexpression of β-catenin and other elements of the canonical Wnt pathway have been observed in samples from patients with AML and other types of hematological malignancies, suggesting that overactivation of this pathway plays a role in the physiopathology of these cancers [43,44,46,47,52,53]. In addition, the bone marrow stroma appears to play an important role in this process; the Wnt pathway is activated in a paracrine manner and the expression of β-catenin is higher in bone marrow AML cells than in peripheral blood. This fact suggests that its activation might come due to signals from the microenvironment [59].

Likewise, several studies reveal a close relationship between the canonical Wnt pathway and LSCs, since its overactivation seems to be involved in the transformation of pre-LSCs into AML LSCs [57,58], as well as in the maintenance of their quiescent state, which confers them with chemoresistance and self-renewal capacity [60,75]. These chemoresistant LSCs would eventually lead to relapse of AML. Therefore, Wnt targeting seems a feasible option to eradicate LSC and reduce the high rate of AML relapses. Nevertheless, we should not forget that crosstalk could occur with other pathways related to LSC maintenance and regulation, such as Hedgehog and Notch, which are also essential in embryogenesis and tissue renewal [80,81].

## 6. Conclusions and Future Directions

The role of the Wnt pathway in embryogenesis and adult tissue renewal has been extensively studied; the activation of Wnt in adult normal hematopoiesis seems to be dose-dependent. Nevertheless, Wnt signaling pathways may be important in hematological malignancies, since the overexpression of β-catenin is observed in AML samples and its overactivation might regulate the quiescence state of LSC. Therefore, delving into the effects of this pathway, along with others such as Hedgehog and Notch, could open a new therapeutic window to chemosensitize LSCs and prevent patient relapse in AML.

In order to avoid relapses in AML, it is important to eradicate the LSCs. One of the current approaches is to target this population; nevertheless, due to the similarities between LSCs and HSCs, it is hard to target the malignant cells correctly. In this review, we have summarized the role of Wnt in both populations to conclude that an overexpression in this pathway is common in AML. Furthermore, we have seen that the activation of Wnt entails an increase in the quiescent state. Both findings seem to suggest that the inhibition of Wnt could be a good therapeutic target to eradicate the LSCs of AML. Furthermore, it may be that the Wnt pathway is not necessary for adult hematopoiesis.

It is likely that the best way to inhibit the Wnt pathway is to target β-catenin. This protein translocates to the nucleus and finally activates the expression of target genes. We believe that more experiments focusing on the inhibition of the correct Wnt signaling pathway, which could decrease the quiescent state of the LSC population to eradicate them with conventional chemotherapy, are required. Following this strategy, we could reduce AML relapses and improve the reduced overall survival of patients with this disease.

## Figures and Tables

**Table 1:** Clinical trials on Wnt/β-catenin-targeted agents in AML. Source: https://www.clinicaltrials.gov/ (accessed on 27 January 2023).

Drug	Mechanism of Action	Clinical Trial	Phase	Status
CWP291	β-catenin degradation	NCT01398462	I	Completed
PRI-724	β-catenin/CBP antagonist	NCT01606579	I/II	Completed
Sulindac	Blocks PDZ domain of Dvl, COX inhibitor	NCT01843179	II	Withdrawn
Celecoxib	COX-2 inhibitor	NCT03878524	I	Recruiting
LY2090314	GSK-3 inhibitor	NCT01214603	II	Completed

## Data Availability

Not applicable.
